# Navigating the unseen peril: safeguarding medical imaging in the age of AI

**DOI:** 10.3389/frai.2024.1400732

**Published:** 2024-12-09

**Authors:** Alexandra Maertens, Steve Brykman, Thomas Hartung, Andrei Gafita, Harrison Bai, David Hoelzer, Ed Skoudis, Channing Judith Paller

**Affiliations:** ^1^Center for Alternatives to Animal Testing (CAAT), Johns Hopkins Bloomberg School of Public Health, Baltimore, MD, United States; ^2^Independent Creative Technologist, Boston, MA, United States; ^3^CAAT Europe, University of Konstanz, Konstanz, Germany; ^4^Radiology and Radiological Science, Johns Hopkins University School of Medicine, Baltimore, MD, United States; ^5^SANS Technology Institute, Rockville, MD, United States; ^6^Department of Oncology, Johns Hopkins University School of Medicine, Baltimore, MD, United States

**Keywords:** medical imaging, artificial intelligence, data quality, precision medicine, data bias

## Abstract

In response to the increasing significance of artificial intelligence (AI) in healthcare, there has been increased attention – including a Presidential executive order to create an AI Safety Institute – to the potential threats posed by AI. While much attention has been given to the conventional risks AI poses to cybersecurity, and critical infrastructure, here we provide an overview of some unique challenges of AI for the medical community. Above and beyond obvious concerns about vetting algorithms that impact patient care, there are additional subtle yet equally important things to consider: the potential harm AI poses to its own integrity and the broader medical information ecosystem. Recognizing the role of healthcare professionals as both consumers and contributors to AI training data, this article advocates for a proactive approach in understanding and shaping the data that underpins AI systems, emphasizing the need for informed engagement to maximize the benefits of AI while mitigating the risks.

## 1 Introduction

Just days after President Biden signed an executive order to protect against threats posed by AI, Vice President Harris announced the formation of an AI Safety Institute, noting that AI also has the potential to cause profound harm. So far, most of the discussion around AI safety has focused around the most obvious risks in areas of biotechnology, cybersecurity, and critical infrastructure (Biden, 2023). However, there are several risks of AI that are unique to the medical community. Cybersecurity threats are already an issue for health care delivery and have already negatively impacted patient care (Neprash et al., 2023). Even cyberattacks that do not adversely impact patient care are costly - for example, the Irish National Orthopedic Register was able to avoid impacting patient care by reverting to a paper-only system, but at a cost of 2,850 additional person-hours for data reconciliation (Russell et al., 2023). The frequency and severity of data breaches are also escalating. In 2021, 45.9 million records were compromised, which increased to 51.9 million records in 2022 - and 2023 set a new and alarming precedent, with 133 million records being exposed, stolen, or otherwise leaked (HIPAA Statistics, 2024). Medical imaging in particular can be vulnerable to malware embedded in common file formats such as DICOM as well as falsification of records (Eichelberg et al., 2020). The US Department of Health and Human Services estimated that vulnerabilities in medical imaging servers were responsible for over 275 million unsecured images across 130 health systems (Bowers et al., 2022).

As AI-driven systems become increasingly integral to medical imaging – from analyzing X-rays and CT scans to diagnosing diseases through dermatoscopic images – the integrity and security of these systems are paramount. An additional challenge is making sure that any algorithm that impacts patient health is thoroughly vetted. Both will have to be addressed to ensure that we can enjoy the benefits of AI while mitigating the risks. Toward this end, ANSI, for example, has designed a new international standard to help organizations use AI systems responsibly, providing a framework for managing AI risks and opportunities across all sectors. The standard outlines a “Plan-Do-Check-Act” approach to ensure the quality, security, transparency, and reliability of AI systems and address concerns, making AI adoption a continuous process (American National Standards Institute, 2023).

While the overall reliability and security of AI systems are obviously a concern, a more subtle threat exists that demands equal attention: the threat that AI poses to itself and the data integrity of our medical image data systems, as well as our broader medical information ecosystem.

It is critical for everyone in the healthcare field to begin thinking about their roles as consumers of AI and as potential creators and curators of the training data that makes AI possible. When confronted with AI approaches, many physicians may envision opaque algorithms beyond their comprehension or critique. However, the most common problems that arise in AI are typically not the result of the algorithm but are instead due to problems with bias in the training data or extrapolation beyond what the training data justifies. Therefore, thinking about how AI training data is generated over time, and how it is used, allows a clinician to be an informed user (versus a passive and potentially dangerous user).

The need to guard against both bias in data sets as well as documenting data sources is addressed by the ANSI CTA-2090 aimed at medical devices (American National Standards Institute, 2021; Nist, 2024). Here, we discuss why data set integrity is particularly critical for medical imaging and the need for physicians to understand the broader issues at stake. We focus on a few key issues - the challenge of ensuring our data sets are both secure and comprehensive; the need to think carefully about the proliferation of medical images captured on diverse devices outside of a clinical settings, such as smart phones; how data gathering can lead to bias, and lastly the challenges generative AI will pose to the integrity of our data sets as well as the already significant problem of medical mis- and dis- information.

## 2 Implications for practice

### 2.1 Data sets are as critical to developing robust AI as algorithms

While most advances in AI are discussed in terms of improved algorithms, such as a deep learning, the reason artificial Intelligence systems can achieve such a high success rate in the prediction and diagnosis of cancer and other diseases is due to the size and quality of their training data. A fundamental requirement for training an effective algorithm is a set of verified training data large enough to accurately represent the population of interest (Chan et al., 2020).

Sun et al. (2017) found that performance on vision tasks (e.g., identifying whether an object is a dog or cat, etc.) increased linearly with orders of magnitude of training data size, continuing to increase even as the training set grew to over 300 million images. The greater the number of images, the better coverage of the problem space. In a 2016 study, Wissner-Gross argued that datasets were likely the limiting factor inhibiting the development of artificial general intelligence (AGI)—not algorithms; over the past few decades, the average elapsed time between key algorithmic advances was about 18 years, versus less than 3 years between key dataset availabilities and advances. In other words, AI breakthroughs were constrained not by algorithmic advances, but by the availability of high-quality training data (Wissner-Gross, 2016). While LLMs (large language models) and transformer models in general have indeed been a significant breakthrough in what AI can do, the fact remains that they depend upon very large data sets.

While vision tasks have benefited from the ImageNet dataset, which can scrape an infinite variety of images from the Web. In contrast, medical images are a limited resource that cannot be produced in the same abundance. Ensuring they are accurately labeled is intrinsically more difficult; ensuring they are “representative” is both more challenging and more important – images of breast cancer, for example, need to include adequate samples of dense tissue and rare tumors. As a result, we are witnessing a gold rush of training-data collection in the race to produce the largest dataset possible, and especially fresh training data (Mah, 2023). This need stands in contrast to the stated desires of patients, 63 percent of whom object to having their data used for AI. Zahlan et al. (2023) some of this reluctance no doubt coming from previous use of personal data for behavioral manipulation by private and public organizations.

Countless image datasets of pathologic and radiologic AI-training data, both public and private, are available online, as seen in Table 1.

**Table 1:** Main medical imaging repositories, data availability and submission and download process.

Repository category / name	Contents	Data provided	Data sources	Security & integration	Submission process	Download process
NIH databases						
NIH clinical center CXR8	45GB of chest x-rays	Labels, annotation, and diagnoses	National Institutes of Health Clinical Center	Box repository	-	Publicly downloadable data
The cancer imaging archive (TCIA)	Large archive of radiology cancer images.	Related supporting data (e.g., patient outcomes, treatment details, genomics, analyses.)	NIH National Cancer Institute	De-identification scripts leverage the DICOM PS 3.15 standard, removes protected info while preserving metadata.	TCIA Advisory Group experts curate, quality control, and de-identify every image collection.	Publicly available archive used for developing and validating algorithms
Genomic data commons (GDC) data portal	A robust platform for cancer researchers and bioinformaticians.	Pathology and radiology images, metadata, and pathology reports	NIH National Cancer Institute Cancer Genome Atlas (TCGA), Emory U. Cancer Digital Slide Archive (CDSA), and TCGA	-	-	CDSA allows browsing of TCGA images, metadata, and reports without login or account.
NIH deepLesion	Diverse repository of over 32,000 CT images.	Medical data from 4,400 patients. Critical radiology findings enhances lesion detection.	The National Institutes of Health (NIH) Clinical Center	Box repository of “thoroughly anonymized” images.	-	Publicly available repository
NIH medical imaging and data resource center (MIDRC)	Over 165,000 medical images to foster AI development around COVID-19.	Imaging studies with clinical data and DICOM tags. Plus, tutorials, performance metrics decision tree, bias awareness tool, and user portal.	National Institute of Biomedical Imaging and Bioengineering (NIBIB). Imaging data and metadata from medical centers, hospitals, and others via the Radiological Society of North America (RSNA), the American College of Radiology (ACR), and via a Data Commons Portal on the Gen3 Data Ecosystem.	Public open data commons created at scale to be interoperable with other data commons via a common query infrastructure.	MIDRC coordinates data access and management at three stages: (1) intake (curation, de-identification, abstraction, and quality assessment) (2) semi-automated image annotation and labeling, and (3) distributed access and query methods.	Free, open-access repository.
National library of medicine MedPix	Over 12,000 patient case scenarios, 9,000 topics, and nearly 59,000 images.	Medical images, teaching cases, and clinical topics, integrating images and textual metadata.	Hosted by the NLM at the Lister Hill National Center for Biomedical Communications in Bethesda, MD	-	-	Free open-access database for health professionals, students, and “anyone seeking medical image data”
Federal interagency traumatic brain injury research informatics system (FITBIR)	An extensible, scalable informatics platform for TBI-relevant data.	MRI and PET TBI images with clinical assessment, environmental and behavioral history, demographics, and biomarkers.	FITBIR contains data records from studies funded by the DoD and NIH.	Subject-level de-identified TBI research data. Facilitates collaboration with labs and informatics platforms.	Two-tiered submission strategy maximizes quality and benefit for investigators.	Researchers request access to data stored in FITBIR.
University databases						
The open access series of imaging studies (OASIS)	Over 2,157 raw imaging scans for data analyses, neuroanatomical atlases, and segmentation algorithms.	Cross-sectional, longitudinal multimodal imaging MRI data. Neuroimaging data sets. PET imaging from tracers, PIB, AV45, FDG.	Accompanying post-processed files from the Pet Unified Pipeline (PUP)	-	-	Freely available to the scientific community.
Stanford artificial intelligence in medicine / medical imagenet.	A petabyte-scale repository of clinical images linked to genomic data and electronic medical record information.	Annotated, de-identified radiology and pathology images.	-	De-identified images. Stanford shares data to foster transparent and reproducible collaboration to advance AI in medicine.	Provides a Review Process Map and a Guide for Sharing Health Data.	Datasets are available to the public to view and use without charge for non-commercial research purposes.
USC stevens neuroimaging and informatics institute image and data archive (IDA)	Neuroscience data on development, aging, and disease progression.	Data collected from 97,128 subjects for 151 studies in 165 countries.	The Laboratory of Neuro Imaging (LONI)	Provides tools for de-identifying, integrating, searching, visualizing and sharing neuroscience data	Investigators maintain data control. Robust, reliable infrastructure protects and preserves research data.	40-page user manual explains the upload and download process.
Johns hopkins diffusion tensor imaging (DTI) / Laboratory of brain anatomical MRI	High resolution MRI scans to facilitate research in DTI data processing and analysis or as control data.	Raw and processed normal population DTI data. Basic imaging parameters provided, with details available.	-	Outdated website with dead links. Embedded unsupported Adobe Flash elements make it vulnerable to attack	-	Open to the public once a user is registered
Hospital-run databases						
Medical information mart for intensive care-CXR (MIMIC-CXR)	227,835 imaging studies from 64,588 Beth Israel Deaconess Medical center patients (2011–2016).	377,110 total images. Includes radiology reports by radiologists during routine clinical care.	Hospital picture archiving and communication system (PACS) in DICOM format.	De-identified images and reports. X-ray analysis linking with clinical data from MIMIC-IV modules.	-	-
Gamified platforms						
Grand challenge	Gamified platform for machine learning solutions in biomedical imaging and algorithm assessment.	Data from 180 medical imaging challenges along with 113 algorithms.	-	-	Users may easily and securely upload medical imaging data.	Users may easily and securely manage data access.
SICAS (Swiss Institute for Computer Assisted Surgery) Medical Image Repository (SMIR) full body CT Scans	Collaboration platform for translational R&D, education, technology, and innovation.	Post-mortem full-body CT scans from 51 subjects.		“Face soft tissue is removed to anonymize the data set.”	-	-
COVID-19 databases						
The UK national COVID-19 chest imaging database (NCCID)	Established by National Health Service User Experience (NHSX). Deepens understanding of COVID-19.	Processed CXR, CT, and MR images plus de-identified DICOM header data and associated clinical data.	UK hospital patients. Collaborates with a national consortium that collects samples and scans from UK Trusts.	De-identified DICOM header data and associated de-identified clinical data stored in AWS S3.	-	NHSX sends approved organizations access to data (AWS credentials) via encrypted email.
COVID-19 image dataset	Hosted by “the largest community of data scientists.” Improves COVID-19 detection via CXR.	-	Public GitHub account of U. Montreal profs, the Radiological Society of North America (RSNA) website.	-	-	-

As shown in Table 1, open-source medical dataset repositories are diverse in their data sources, their approach, and their execution. They range from neglected sites that contain dead and insecure pages to robust (even gamified) platforms that provide manuals, tutorials, and supplementary materials, that crowdsource new algorithms via a programmatic interface (REST API), and that require a submission process that takes months to complete (NIH National Cancer Institute CIP Cancer Imaging Archive, 2024). In some cases, viewing and downloading images and metadata (clinical data, DICOM tags, pathology reports) are immediately available to anyone, while other repositories require accounts not just for submission but also for data access. Some collections are static while others are updated. The vast abundance of medical imagery comes at a cost to accuracy, standardization, and security. The most obvious problem occurs when cross-pollination of disparately pre-processed data between platforms, which typically results in AI inaccuracies, while inadvertent duplication of training data leads to AI bias and inflated accuracies. Data sets for other areas of precision medicine are equally diverse in terms of sources, curation, and scope, making benchmarking a challenge (Abbaoui et al., 2024; Nist, 2024). The gold standard for training data sets is manual annotations, however these can have significant variability between physicians. This has been mitigated by attempting to use multiple raters or other types of quality control, yet inaccuracies can remain - a review of two neurosurgical datasets (RESECT and BITE) identified inconsistencies which would likely impact the validity of algorithm evaluations (Luo et al., 2022).

One example comes from the early days of the pandemic when a COVID-19 study from University of Cambridge researchers achieved less-than-ideal results. Roberts and Driggs set out to explore deep-learning models for diagnosing COVID-19 and predicting patient risk by analyzing CXR and CT scans (Roberts et al., 2021). But after reviewing over 400 tools, they concluded none reached the thresholds necessary to support clinical use—primarily due to poor-quality (garbage-in) datasets, methodological flaws, and risk of underlying bias. Approximately half of the data came from public datasets. The most common reason for exclusion was the lack of adequate data description and poor documentation of pre-processing, as the study’s inclusion/exclusion criteria included requiring documentation of any resizing, cropping, and normalization of images that occurred prior to model input.

Many public datasets contain pre-processed, low-resolution, or compressed preprints (e.g., JPEG, PNG, or similar versus DICOM format). This leads to quality loss while impacting consistency and comparability. Resolution loss is a particular concern when it is not uniform across classes. The lack of DICOM metadata inhibits our understanding of the impact of image-acquisition parameters (e.g., scanner manufacturer, slice thickness, etc.) (Roberts et al., 2021). At the same time, DICOM data may not be ideally suited for the large-scale and ubiquitous use of AI as it was recently found that the meta-data did not adequately protect patient privacy (Bushey, 2023).

Recently, Holste et al. (2023) offered similar words of caution about the risks that preprocessing, specifically “pruning,” poses on the integrity of images stored in medical repositories and on the accuracy of diagnoses derived from pruned images. Though pruning is a powerful compression method that reduces memory use and inference time without significantly impacting the overall performance of a deep neural network, Holste et al. (2023) warn that the nuances of how pruning impacts model behavior are not completely understood, especially when it comes to the long-tailed, multi-label datasets common to clinical settings. Further, they predict this knowledge gap might yield unexpected model behavior that impacts patient well-being should a pruned model be used for diagnosis (Holste et al., 2023). In the first analysis of pruning’s effect on neural networks trained to diagnose thorax diseases from chest X-rays (CXRs), Holste et al. (2023) examined which diseases are most affected by pruning on two large CXR datasets. They identified CXRs where uncompressed and heavily pruned models disagreed (known as ‘pruning-identified exemplars (PIEs)’) and found that radiologists perceived PIEs as having increased label noise, poorer quality images, and greater diagnosis difficulty (Holste et al., 2023). Similarly, as seen earlier in the study from Roberts et al. (2021), pre-processing and compressing images into non-DICOM formats resulted in losses in quality, consistency, and comparability. This issue is most obviously a concern for radiology where data-rich images create high demands for data storage infrastructure as well as data processing, but as AI seeks to use disparate sources of data, some thought will have to be given to the costs and benefits of different types of data compression and pruning.

### 2.2 The challenge of filters and other sources of bias

As AI is democratized into handheld devices, there is another potential concern that is especially acute for dermatology but will likely be an issue for other fields going forward. Scientists have already devised a deep learning model that may be used with smartphones as a decision support mechanism for dermatologists and clinicians (Yilmaz et al., 2022). This is vital as more and more patients interact with caregivers via telehealth, and these interactions will only increase as our smartphones will be equipped with more and more sensors, providing data that will be used for AI training purposes. However, there is an additional concern about the integration of AI filters into smartphone cameras (computational photography) - Samsung Galaxy owners have complained their AI-enabled cameras are swapping out zoomed shots of the moon with a canned image and giving their babies teeth (Kritsonis, 2023; Schneider, 2023). Apple’s new Smart HDR (high dynamic range) features are less reality-distorting but still matter (perhaps even more so, since the effects are less obvious) (Smith, 2021). The ubiquity of these new tools may also pose a threat to the integrity of images submitted to care providers, especially if these images are used for training data. And chances are they already have—since these tools have been manipulating our phone images longer than many realize (Lu, 2019). Unfortunately, the variation of effects across brand, and the evolution of these filters over time could also change our AI’s ability to correctly identify and diagnose lesions and other skin conditions, especially since manufacturers likely update their algorithms regularly but do not make this information public. There is, however, an easy fix – imaging data used for both diagnostics and training data should always be made with an app that specifically does not use filters, AI or otherwise, in essence creating a “medical mode “for the devices, and training data should be curated to ensure it features only unfiltered data.

In conclusion, the Roberts et al. (2021) study advises caution over the use of public repositories moving forward, both because of source issues and ‘Frankenstein’ datasets (datasets assembled from other datasets and redistributed under a new name) which can compound the risk of bias and dataset “peaking” caused by the duplication of data. Dataset repackaging inevitably results in the training and testing of algorithms on overlapping or identical datasets which are incorrectly assumed to be derived from unique sources (Roberts et al., 2021).

Subtle biases can creep in as well. For example, AI algorithms may exhibit bias when interpreting CXRs by associating more severe disease with specific chest radiograph views (e.g., an anteroposterior view is commonly used for immobile patients versus a standard posteroanterior projection). Overrepresentation of severe disease also impacts clinical utility since diagnosis of disease in its early stages offers greater benefit. Finally, the regular updating of publicly available datasets has the potential to create further ambiguity by inhibiting replication of published results. Going forward, any clinical AI applications should have a cached version of the dataset or at the very least, documented the date/version of data used.

To facilitate adoption into clinical practice, Roberts et al. (2021) also stressed the necessity of enabling interpretability of the model by describing features that influenced the model, by overlaying a saliency map on the image to indicate those features, by linking prognosis to underlying biology, and by identifying patients with similar clinical pathways – clinically useful AI must be anchored in biological mechanisms in a way that tasks such as image recognition do not. While there may always be a trade-off between explainability and performance, explainable AI is essential to understand the reasoning behind model decisions and to facilitate generalizability across diverse patient populations and clinical settings (Markus et al., 2021). The authors concluded that existing models designed for diagnosing radiological imaging data are hampered by the quality of their training data, that current public datasets are inadequate in terms of size and quality for training reliable models, and that any studies leveraging these datasets will exhibit a high (or unclear) bias risk. In the end, none of the machine learning models in their review were identified as candidates for clinical translation for the diagnosis/prognosis of COVID-19. The authors of the study call on researchers worldwide to improve the size and quality of these datasets by submitting their data for public review (Bachtiger et al., 2020). The authors of this article suggest that in addition to public review, the data’s origin must also be made known to ensure its authenticity and integrity, whether via a digital signature or via the use of blockchain technologies. This is consistent with the National Institute of Standards and Technology (NIST) initial statement on global standards for AI, which included a mechanism for enhancing awareness and transparency about the origins of digital content (Nist, 2024).

While Roberts et al. (2021) assert that many papers used in their study failed to note the original source of their images, they state that about one-third of the public-repository data came from mainland China, with additional data coming from France, Iran, the US, Belgium, Brazil, Hong Kong, and the Netherlands. The openness of public datasets made it impossible to determine if patients were truly COVID-19 positive or had underlying selection biases – in other words, the models were potentially being trained on inaccurately labeled data. Many public repositories allow anyone to contribute images, with no restrictions (Roberts et al., 2021). This openness comes with risks, however – from carelessness or intentional disruption.

### 2.3 Datasets needs to be guarded against generative AI and nightshade

It would not be difficult for a bad actor to taint a set of images (even DICOM images) with non-visual alterations that cause a trained diagnostic model to misbehave, influencing a public dataset to have a downstream impact on the networks trained on that data.

One potential threat comes because of our attempts at rights protection using techniques such as Nightshade, a tool meant to empower artists and protect their work from being harvested by AI without their permission (Drost, 2023). There is concern that Nightshade could seriously damage image-generating AI models by altering training data through the insertion of invisible pixel changes (Heikkilä, 2023). According to one study, the ingestion of just 50 corrupted images is enough to cause distorted results; 300 Nightshade images will corrupt an image generator entirely (Shan et al., 2023). The effect of Nightshade on generative models is instructive since these techniques will have a similar detrimental effect on the efficacy of an otherwise useful model trained for diagnostic purposes.

Optimized, prompt-specific data poisoning corrupts the AI training data. In other words, Nightshade circumvents the problem of large AI datasets by targeting the prompt. According to *Decrypt*, the easiest way to deceive an AI model into thinking a cat is a dog is simply by mislabeling a few hundred images of a cat as a dog; a sufficient number of attacks will render a model worthless, meaning that one route of attack on our medical image repositories could be the use of Nightshade to destroy the AI’s ability to accurately identify, categorize, diagnose, and understand diseases (Nelson, 2023).

In theory, Nightshade could also be used by medical imaging device manufacturers to introduce a specific type of artifact, like Nightshade, into public image sets or surreptitiously into clinical images. While not affecting the clinician’s ability to make a human diagnosis, such artifacts would cause a competitor’s AI solution to fail, which could lock customers into licensing a corporation’s diagnostic agent. Given the centrality and importance of high quality, accurate data for the future, it is important to start thinking about how we want to manage the ways in which corporations control and profit from such data.

Generative AI poses another threat. There are several ways to weaponize generative AI. Not only could it be used to spoof a single sample, it could potentially also be used to spoof entire data sets, repositories, or even scientific organizations. Despite attempts to implement guardrails, several popular LLMs will fabricate fake scientific references in support of dubious health claims (Menz et al., 2024).

Thus, AI models used clinically must be strongly protected from end to end, above and beyond the normal cybersecurity concerns. The AI’s training data, ingestion pipeline, inter-stage pipelines, and the model itself must all be protected.

In fact, it is a significant problem when AI is trained on its own data. As larger and more complex models require more data, there is a temptation to use synthetic data or to use AI to make human annotation (currently the gold standard by which models are judged) more efficient and productive. However, this can form an echo-chamber, magnifying the bias of an algorithm. It’s been shown that data precision and diversity progressively degrade over generations when an autophagous (Greek for “self-devouring”) loop fails to be supplied with adequate fresh real data. This has been coined Model Autophagy Disorder (MAD) (Alemohammad et al., 2023). We’ve already witnessed several real-world examples of bad AI-generated answers forming online feedback loops of misinformation. First, because AI models lacks constraints that limit possible outcomes, they sometimes “hallucinate” facts, being led astray by its probability-based language models into coherent but incorrect statement (AI hallucinations?, 2022). When hallucinated content gets indexed by Google, AI-generated misinformation can even pollute search engine results – as happened when ChatGPT hallucinated that eggs can be melted, and the result was indexed on Google search (Edwards, 2023). When Microsoft replaced news editors with AI, the results were disastrous. Suddenly, the default Microsoft start page featured false news stories from dubious sources (Breen, 2023). If AI feedback loops and disinformation can prove problematic for Google and Microsoft, consider the damage that intentionally false metadata, misleading saliency maps, and erroneous pathology/radiology reports could cause to the accuracy of our AI training.

However, somewhat contradictory, synthetic training data can improve the performance of large language models (LLMs) in various tasks. Research has shown that while models trained on human-labeled data often exhibit superior performance, synthetic augmentation can be beneficial, especially in enhancing performance on rare classes within multi-class tasks (Møller et al., 2023), and this could certainly be useful for diagnostic algorithms. Additionally, synthetic data generation through fine-tuning of teacher LLMs has been found to significantly enhance downstream model performance in text classification and text generation tasks, sometimes requiring only a small fraction of the original training dataset (Kaddour and Liu, 2023).

### 2.4 AI can contribute to misinformation and disinformation

Despite AI’s impressive success rate at interpreting radiological and pathological images, the type of AI that people are most aware of is Large Language Model (LLM) systems such as ChatGPT. Patients will increasingly depend on them for quick searches about medical information and treatment options. Clinicians will, in the future, depend on them for literature searches, will use AI-enabled systematic reviews to guide clinical practice, and AI-facilitated note taking for clinical reports. As foundational models become more elaborate, they can extend beyond text-prompts to much more complicated decision-making support based on a combination of both text and image inputs (Azad et al., 2023).

Despite their impressive achievements, LLMs and other foundation models do not make great substitutes for humans when it comes to offering cancer treatment information. A recent study found the AI hallucinated incorrect suggestions for localized treatment of advanced disease, targeted therapy, or immunotherapy in 13 of 104 (12.5%) of cases (Chen et al., 2023). A full one-third of AI-recommended treatments were non-concordant with National Comprehensive Cancer Network (NCCN) guidelines. The study confirmed that while LLMs can provide better diagnoses than the average person, the chatbot failed to provide accurate cancer treatment recommendations and tended to combine false recommendations with correct ones—making discerning the truth difficult—even for experts. Here, too, data sabotage or simply a flood of misinformation can prove damaging if more patients turn to LLMs or their extensions to interpret their medical data. It has been recommended that clinicians make sure their patients understand that LLM chatbots are not reliable sources of treatment data (Chen et al., 2023). This implies the authors believe AI will pose an increasingly greater risk as more patients turn to LLMs for medical advice. Thus, data sabotage could also become a more significant risk. The idea of intentionally flooding our LLMs with false medical information to harm a population is not out of the realm of possibility; disinformation about AIDS was deliberately spread by the former Soviet Union and significantly hampered the public health response in many countries (Geissler, 2016). Given the known attempts on the part of many state and non-state actors to manipulate the information ecosystem, it’s not a far cry to assume similar disinformation campaigns might attempt to target and corrupt our AI training data or, alternatively, AI generated text and images can begin to subtly subvert LLMs (O’Sullivan et al., 2023). Some LLMs, when prompted, will concoct convincing sounding health misinformation [i.e., that sunscreens cause cancer (Menz et al., 2024)]. In this respect LLMs, have been termed weapons of mass health disinformation. Generative AI can now produce convincing patients, radiology images with generative AI tumors, and clinical data in the service of disinformation.

People are, for better or worse, inclined to be strongly influenced by appearances, which may not accurately reflect reliability or risk. This can result in a side effect of technology known as “overtrust,” where individuals habitually accept software recommendations without critically evaluating the situation (Hardré, 2016) and physicians are not immune to this risk (American National Standards Institute, 2023).

Ultimately, our LLMs merely distill our collective research output – which should give us pause about current trends in the biomedical literature The number of papers retracted has grown substantially as a portion of published literature, with more than 10,000 research papers retracted in 2023 - and this likely does not include the many papers published (often assisted by AI) in paper mills, nor the extensive preprints that can contribute substantially to misinformation (Brierley, 2021; Noorden, 2023). Therefore, in addition to image datasets, the larger body of clinical literature will also likely serve as training data for clinically impactful LLMs going forward, and we must give some thought about how we ensure this process does not go awry.

### 2.5 Actionable recommendations

Datasets require standardization and documentation: this will include the standardization of data formats and documentation across medical dataset repositories, including the standardization of language used to describe all image pre-processing, resizing, cropping, and normalization techniques, as well as the use of AI image filters, and the standardized formatting and encoding of this information in the image metadata. Guidelines should require comprehensive documentation of diagnostic labels. Quality assurance should include ensuring the data set does not include products of generative AI.

Data sets from computational photography in mobile devices should use a medical mode or app that ensures filters are by-passed. Additionally, the appropriate metadata for submission to a health care provider or medical image repository would be appended to the image to provide the most consistently formatted results possible across all brands and devices.

Security is critical: this can include access controls for sensitive medical data, the use of blockchain for authentication, and balancing the trade-off between immediate access and controlled access for some data sets. Steps should be taken to ensure watermarks such as Nightshade do not poison a dataset.

Datasets must be representative: this means including as wide a range of patients and clinical scenarios as possible –this includes considerations of age, gender, and other obvious factors that can lead to bias. However, it should also include a commitment to gathering data in real-world clinical settings to capture natural variability and complexity. Active bias mitigation measures must be ongoing.

AI will be part of everyone’s information ecosystem, and it must be guarded against misinformation and disinformation. This will require implementing safeguards against the intentional flooding of LLMs or other models with false medical information and enhancing the resilience of AI models against adversarial attacks.

Cross-analysis of image data with all other image repositories will need to occur to ensure data has not been duplicated elsewhere and does not include the products of generative AI. The most effective way to accomplish this may be via the creation of secure national centralized storage centers, or, at least, the creation of a centralized platform for searching, submitting, and accessing all existing medical image data. Absent of this, the global standardization of medical image data could be achieved by private enterprise—as a service offering by a company that specializes in medical image data processing for the generation of AI training data.

All stakeholders should be educated about AI. Standardized training programs, offered in science programs and medical and nursing schools around the world would help ensure all stakeholders are sufficiently educated about AI. A formal legal consent system should be put in place for the use of medical images as training data—not just x-rays and MRIs, but published case reports and notes in electronic health records. Scientists and physicians need to be mindful that they are actively producing training data – not just with each chest x-ray, but with their published case reports and notes in electronic health records. Even their own questions posed to ChatGPT or other LLMs will be used to train future algorithms. Therefore, everyone in the medical community has a responsibility to be an informed participant in the development and use of AI. Future work should include surveying physicians to ascertain understanding of how AI training data is generated and their role in this process, research into understanding how physicians can be alert to problems in AI generated recommendations, how data security can be implemented at every level - research oversight committee including IRBs should consider monitoring data generation, data integrity, and data use for routine clinical trials as well as registry.

## 3 Discussion

The benefits of AI are obvious: as early as 2017, machine learning algorithms used in the prognosis of skin cancer were found to have an accuracy rate equal to a dermatologist (Esteva et al., 2017). One recent study found that AI models are now able to classify brain tumors in MRI images with an accuracy rate of 98.56% (Chan et al., 2020). AI applications in oncology now include risk assessment, early diagnosis, patient prognosis estimation, and treatment selection. AI has enormous potential for boosting accuracy and improving outcomes when it comes to predicting certain types of breast, brain, lung, liver, and prostate cancer and has proven highly accurate at predicting recurrence and, for colorectal cancer, risk stratification with higher accuracy than current guidelines (Liu et al., 2019; Nartowt et al., 2019; Bębas et al., 2021; Zhang et al., 2023).

AI promises faster, less expensive, and more accurate processing than humans are capable of, a more detailed analysis of tumors, facilitated exploration of potential therapies, and better -informed decision-making. The medical community increasingly relies on AI as a diagnostic partner for interpreting pathology and radiology images. AI therefore has the potential to be a ‘force-multiplier’ for physicians, although whether this is implemented equitably to increase medical access for all, or whether it evolves into a two-tier system where only the wealthy have access to ‘helpful AI’ with input from a ‘non-AI’ doctor will depend on what are sure to be difficult policy decisions. One small but critical step in this process is thinking through the implications of how medical image data is collected, how the data is maintained, curated, and authenticated—and most importantly, who controls the access to that data.

As a result of being built-up piecemeal over the years by independent parties with little intercommunication, our medical image repository ecosystem has become a mottled sea populated by siloed islands. The disparate methods each repository follows for accepting, processing, formatting, storing, indexing, interpreting, and providing image data (along with any accompanying metadata) can lead to unnecessary AI inaccuracies and bias. Additionally, the lack of standardized methods leaves our repositories vulnerable to infiltration with Nightshade, generative AI, or general disinformation, any of which could corrupt our AI training data and impact the AI’s ability to function properly. Further, the lack of standards makes it impossible for a user to distinguish between a highly vetted, curated repository and one created haphazardly or using generative AI.

Although we focused in this paper primarily on issues of data set quality and infrastructure, it should be noted that AI will present significant legal challenges - for example, in the US, data privacy is regulated under HIPAA; FDA regulates AI as both a device and a product; while the FTC would be responsible for fraud or unfair business practices, which would include bias in outcomes or faulty medical advice from an LLM (American National Standards Institute, 2021). The EUs GPDR has more explicit protection for patients to opt out of having their data used, but the EU’s AI Act only grants individuals the right to explanation of decision-making (Article 86) - but this excludes AI medical devices (van Kolfschooten, 2024). Overlaid upon this in many jurisdictions will be the regulatory bodies of medical societies as well as licensing boards. Despite this, there are many aspects of AI that should be addressed both legally and by stakeholders. HIPAA was not intended to safeguard patients from being used as training data unknowingly, nor does any professional medical body have standards for the terms under which physicians are expected or required to produce training data. No legislation currently specifically targets data poisoning or AI-assisted disinformation.

However, there are steps that can be taken to ensure we have high quality, publicly available data in the future. We call for the establishment of strict universal guidelines and standards to secure our medical image repositories, to formulate consistency of all input and output data, and to control the methods with which the repositories function. Additionally, to help protect the integrity of our AI training data we call for watermarking, not just of all material generated by AI, but also of material that has been treated with Nightshade (or like technologies).

Fundamentally, if algorithms are going to impact clinical care in any meaningful way, it is crucial that the training data used to develop them undergo a much higher level of scrutiny and security than has hitherto been applied. In place of haphazard datasets, there needs to be an investment in large, high quality, and accurately labeled data sets, and this effort should be seen as crucial scientific infrastructure. As data for AI is generated in a more distributed manner – through smartphones and other sensors – it is equally crucial to ensure that this data is as high quality and unbiased as possible. Lastly, ensuring that these data sets are diverse, representative, and available for the public good is critical for enabling AI to deliver on its promise of providing more efficient health care for everyone. Misinformation and disinformation have been a long-standing problem in medicine, as have (to a lesser extent) insufficiently representative datasets and models. AI, however, will present new challenges that differ both in kind and scale. While it is challenging to regulate AI or hold its progress (Thomas, 2023), best practices and standards as suggested here can limit these threats. The time to start addressing these challenges is now - an ounce of prevention will be worth a pound of cure.
